# Views from the borderline: Extracts from my life as a coloured child of Deaf adults, growing up in apartheid South Africa

**DOI:** 10.4102/ajod.v8i0.473

**Published:** 2019-04-24

**Authors:** Jane Harrison, Brian Watermeyer

**Affiliations:** 1 Disability Studies Division, Department of Health and Rehabilitation Sciences, University of Cape Town, Cape Town, South Africa

## Abstract

**Background:**

Over 90% of Deaf parents have hearing children, but there are very few, if any, studies that have explored the life worlds of hearing children of Deaf adults (CODAs) in South Africa. This article is an account of part of the life experiences of a female hearing child who was born and raised by her Deaf parents in apartheid South Africa in the 1980s.

**Objectives:**

This study used auto-ethnography to explore the socialisation of a female coloured CODA during the height of South Africa’s apartheid era, in order to shed light on intersectional influences on identity and selfhood. The study was intended to contribute to the limited knowledge available on the life circumstances of CODAs in Global South contexts.

**Methods:**

Evocative auto-ethnography under a qualitative research paradigm was used to explore the life world of a now adult female hearing child of Deaf parents. Her thoughts, observations, reflections and involvements are articulated in a first person written narrative that is presented in this article. A thematic analysis approach was used to analyse data, and the themes that emerged are: (1) CODAs as language brokers, (2) being bilingual and trilingual, (3) being bicultural, (4) role reversal and parentification and (5) issues of identity. A discussion of these themes is interwoven with the literature, in an effort to provide a rich and robust analysis that contributes to the body of knowledge.

**Results:**

Multiple identity markers that include disability, gender, race, age, nationality, culture and language intersect to frame the life world of a hearing child of Deaf parents who grew up in the apartheid era in South Africa. The result is both positive and negative life experiences, arising from being located simultaneously in both a hearing and Deaf world.

**Conclusion:**

This study suggests that, in part, the life world of a hearing child of Deaf parents is multi-layered, multidimensional and complex; hence, it cannot be presented with a single description. Recommendations that inform policy and practice are outlined in the concluding section of the article.

**Keywords:**

Deaf parents; hearing child; CODA; identity; apartheid; South Africa.

## Introduction

There is a paucity of studies that explore either the childhood or adulthood experiences of children of Deaf adults (CODAs), particularly within African contexts, including in South Africa. One of the very few studies available, by Moroe and De Andrade (2018) set within the context of Gauteng Province in South Africa, highlights the lack of knowledge on the interpreting roles of CODAs in Deaf-parented families. Arising from an auto-ethnographic study of her life and written with the support of her research supervisor, who is thus a co-author of this article, this article is framed around the biography of Jane, a child of Deaf parents. In this section, we start off by defining the terms that form a primary part of the discussion of this article, namely Deaf, CODA and identity.

Some writers state that ‘uppercase D’ in Deaf is used to describe people who identify as culturally Deaf and who are actively engaged in the Deaf community where they use a shared sign language (Padden & Humphries 1998; Reagan 1995). The same authors state that ‘lowercase d’ denotes the medical condition of having hearing loss, and people who identify as deaf with a lowercase ‘d’ do not commonly have a strong connection with the Deaf community and they often do not use sign language, as they prefer to communicate orally. However, some people with moderate hearing loss may choose to identify themselves as Deaf and to be actively involved in the Deaf community, and people who have very limited or no hearing at all may prefer to identify themselves as deaf as opposed to Deaf. As noted by Marzina (2017), a person can be deaf without being Deaf and vice versa. There is therefore no standard definition which practically relegates relevant people to one category or the other, and hence the best way is to go by the definition that a person chooses for him or herself.

‘Children of Deaf adult’ (CODA) is an acronym that refers to a hearing child born to one or two Deaf parents. Although the term has widely been used in the Global North, it is fairly new to South Africa. Statistics indicate that only 4.4% of children born to Deaf parents are also Deaf (Mitchell & Karchmer 2004); hence, a much larger percentage of children of Deaf parents are hearing. Another common phrase used by hearing children born to Deaf parents in referring to themselves is ‘Mother father Deaf’ (Clark 2003). The term seeks to affirm their identification with, and belongingness to, the Deaf community. Representing a linguistic and cultural minority group, CODAs often grow up as part of the Deaf community, acquiring, among other things, Sign Language as a first language (Bishop & Hicks 2005). Closely linked to CODAs is the issue of identity, which is articulated below.

Identity can be viewed as a multi-faceted and evolving phenomenon that a person develops over time about who they are. It includes aspects that they have no control over, such as the colour of their skin, as well as volitional aspects such as the choices that they make in life, including how they spend their time and what they believe in (Ellis, Adams & Bochner 2010). The same authors state that it is not uncommon for people to wonder where they fit in and to ask themselves the questions: ‘Who am I?’ and ‘Do I matter?’. Identity formation may be a complex experience for CODAs, as they are part of the large hearing community, and with their parents, they may be simultaneously part of a Deaf community with different norms and standards. For CODAs, decisions about selecting behaviours and changing self-representations can be very challenging. In order to situate Jane’s narrative in the context of the study, we discuss the sociopolitical setting of apartheid South Africa in the following subsection.

### Sociopolitical context

South Africa’s colonial history began in 1652 with the arrival of Dutch settlers; the colony changed hands several times, before the establishment of British rule in 1806 (Adhikari 2006a). Embedded, structural racism became a legal stipulation with the introduction of the apartheid system under the National Party in 1948 (Trotter 2000). Consequently, the multiplicity of laws separating racial groups into immensely unequal lifestyles required that all South Africans be classified into defined racial categories. The apartheid regime created, for the purpose of segregation, the categories ‘white’, ‘black’, ‘coloured’^1^ and ‘Indian’ (which later became ‘Asian’), and laws that entrenched white supremacy over other races were promulgated (Trotter 2000).

One can argue that racial discrimination and the separation of communities also affected people with disabilities, including Deaf people. Laws such as the *Group Areas Act* (No. 41 of 1950) (Adhikari 2006b) and the so-called homelands policy (Butler, Rotberg & Adams 1978) of restricting all but white people to designated rural areas led to a fragmentation of Deaf communities along racial lines. The *Group Areas Act* also meant that non-white people were relocated, often by force, out of urban areas into townships (Adhikari 2006b). One of those areas where people deemed ‘coloured’ were relocated to was the Cape Flats, a wind-ravaged, sandy area outside Cape Town (Trotter 2000). ‘Coloureds’ in the Cape descended from slaves, the indigenous Khoisan people, and intermarriage between early white settlers, indigenous Africans and Asian slaves (Adhikari 2006a). Even after 23 years of non-racial democracy, apartheid categories still reflect in economic inequalities, and they also remain prominent in how South Africans view themselves and one another.

The following section outlines the methodology that was adopted in undertaking this study, including the data analysis approach. Thereafter, ethical principles that were observed throughout the study are outlined, followed by a discussion of the five themes that emerged from a thematic analysis of Jane’s reflexive journals: (1) CODAs as language brokers, (2) being bilingual and trilingual, (3) being bicultural, (4) role reversal and parentification and (5) issues of identity. Presented as interrelated segments of Jane’s whole life narrative, the themes are embedded with thought-provoking zones of overlap which, as mentioned in the abstract, are interspersed with the literature in an effort to provide a rich and robust analysis that meaningfully contributes to the body of knowledge.

## Methodology

This study used a qualitative evocative auto-ethnographic approach, which represents a combination of elements of both autobiography and ethnography, to draw the life narrative of Jane within a context of South Africa. Auto-ethnography is a research and writing approach that defines and analyses personal experience of the researcher and that also seeks to understand cultural experiences (Ellis et al. 2010). When one is writing an autobiography, the author retrospectively selects and writes about his or her past experiences, thereby challenging traditional research approaches that often seek to speak on behalf of other people (Ellis et al. 2010). When a researcher does ethnography, he or she studies a culture’s beliefs, values, relational practices and shared experiences, with the aim of assisting both cultural members and cultural outsiders to better understand the culture (Maso 2001). Auto-ethnography has also been defined as a personal narrative of one’s own location in relation to others within cultural, economic, political and social contexts (Spry 2001). This study explored Jane’s life experiences of being a CODA within a ‘coloured’ community of apartheid South Africa. To enhance the scientific rigour of this study, we have therefore used both emic and etic perspectives. We have taken Jane’s own views about her own experiences into account (emic) as well as drawn upon external perspectives in the form of literature and theoretical frameworks to describe and interpret the data (etic).

Among the various forms of auto-ethnography, Jane chose to use evocative auto-ethnography, thereby enabling her to self-introspect on the subject, and to allow the readers to connect with her experiences and feelings as both the researcher and participant. The heart of evocative auto-ethnography lies in the ability of the researcher to intimately narrate and analyse his or her own narrative in relation to a particular subject (Mcllveen 2008). Evocative auto-ethnography permits the author to view him or herself as the phenomenon and to write an evocative narrative which is distinctly focused on his or her academic study and personal life (Ellis, Adams & Bochner 2011). That is not to say that the researcher just writes about his or her personal experiences, but it is to say that the researcher is critical about such experiences, in the context within which the study develops (Méndez 2013). According to Ellis (2007), evocative auto-ethnography involves a back-and-forth movement of the researcher between reporting his or her own experiences, examining his or her own vulnerabilities, and at the same time illuminating the wider context within which the experiences took place, in an almost therapeutic way, as further discussed below.

### Data analysis

Auto-ethnographic writing is embedded with some kind of self-reflexive analysis (Ellis et al. 2010); hence, by writing the narrative in the first person, as illuminated in the findings section, Jane was at the same time engaged with data analysis. In addition, a thematic analysis of Jane’s free writings over a period of 3 years (2015–2017), in a hard copy reflexive journal, was also conducted, through reading, re-reading and manually coding data that are relevant to the topic of the study, and pulling together the codes into themes. Thematic analysis is a method of identifying, analysing and reporting themes and patterns within data (Braun & Clarke 2006). Each of the five themes that emerged from an analysis of the writings in Jane’s reflexive journals will be discussed later on in this article, under the section which presents and discusses the findings of the study.

### Ethical considerations

Ethical approval was sought from the institution of study, the University of Cape Town (536/2017). Informed consent was obtained from family members who feature in the biography, albeit without generating data from them. The principle of no harm to participants was considered for Jane who is both the researcher and participant in the study, as well as additional persons who are mentioned in the story but are not participants. Confidentiality was upheld by concealing names of additional characters who are ‘active’ or ‘passive’ in the story. Considering that there is a paucity of studies of the life experiences of CODAs in South Africa, this study represents a voice for other CODAs who did not participate in the study but who may share more or less similar experiences. In addition, the study makes a significant contribution to the body of knowledge and it offers recommendations that inform policy and practice.

## Findings of the study

The findings of this study are primarily the narrative of Jane, which is presented and discussed below, beginning with the subheading ‘My life: An introduction’. Thereafter, the story unfolds under five main themes that emerged from both Jane’s reflexive analysis while writing the story and thematic analysis of her reflexive journals. We mention the five themes again, so as to refresh the memory of the reader on what they are: (1) CODAs as language brokers, (2) being bilingual and trilingual, (3) being bicultural, (4) role reversal and parentification and (5) issues of identity. As previously mentioned, the narrative is written in the first person but it is also interwoven with the literature, in an effort to strengthen the analysis, thereby contributing to the existing body of knowledge.

### My life: An introduction

I am the eldest of two children born to Deaf parents in Cape Town, South Africa, and just like my sister, I am hearing. We both sign and South African Sign Language (SASL) is our first language. On my father’s side of the family, a few relatives can sign, but most if not all of my mother’s family members cannot sign, meaning that her family is not able to communicate with her in appropriate ways that suit the nature of her impairment and that of her husband (my father), and that is where I, a CODA, fit in. When I was below the age of 5, and when most toddlers were exploring their life worlds and playing with peers and toys, I was being trained to become the mouthpiece and ears of my parents. That meant assuming some form of adulthood roles and responsibilities in ways that affected my life in both positive and negative ways.

Remembering my early childhood years in South Africa, I think about a Drum magazine issue of September 1983, which carried a story of a young girl and her Deaf parents, under the caption ‘a little happiness in a silent world’. The story depicts the girl as a ‘bouncing ball of happiness’, alongside comments that allude to the fact that while the parents live in a ‘world of silence’, they are ‘compensated’ by the birth of their ‘perfectly normal’ daughter. I feel that when I was a child, people had the same views about me, in a scenario where a CODA often assumes the role of sign language interpreter at an early age. The narrative of the girl in the magazine indicates that she was also described by society as some form of ‘emotional support’ or ‘compensation’ to her ‘damaged’ parents – a depiction which carries both disablist ideas about deaf people, and a weighty emotional duty for their hearing children.

#### Coloured Affairs, 1981

As an adult, I found myself walking along the long corridors of the Civic Centre in Parow, a working class Cape Town neighbourhood, when a familiar stench penetrated my nostrils, awakening perplexing memories and emotions from decades before. Suddenly, I was a small child walking down the corridor of the Coloured Affairs office. We approach a desk where a plump, stern-faced woman asks, ‘Ja, hoe kan ek help?’ (‘Yes, how can I help?’). I look at my mother who signs to me ‘tell her why here, need help why, problem with disability have’. I relay the message to the woman behind the desk and almost immediately the expression on her face changes, from stern to ‘Oh shame!’ (a South African expression of sympathy).

Her voice is filled with pity as we are handed a piece of brown paper with our ‘unique’ number and shown to our seats. I could hear the murmuring voices of agitated people waiting to be helped, along with the subtle whispers of ‘shame’. Everyone stares at us, rows and rows of coloured people waiting for their number to be called. The stench I recall is what I refer to as the ‘poor Coloured smell’ – a mixture of poverty, stale tobacco and smouldering fire, coupled with the smell of weak tea and peanut butter. The smell brings emotions from a deep-rooted place, and that day in Parow the smell left me feeling sick to the core. I stayed home from work for the next 3 days to avoid having to go back there. It became clear to me that this was one of the many distressing memories that I have tried to suppress over the years.

From my own perspective, ‘procedures’ for ‘determining’ one’s race under apartheid were nonsensical. According to the infamous ‘pencil test’, if a pencil placed in one’s hair did not fall out when released, this was evidence of belonging to the ‘black’ race. Despite the fact of this ‘in or out’ categorisation, the coloured identity itself has been associated with ambiguity (Petrus & Isaacs 2012). A discourse that classifies ethnicity and race has a great role to play in the production of certain kinds of identities, and when such identities are provided with everyday meanings, they become real (Erasmus 2001a, 2001b). Coloured-ness is viewed by some people who self-identify as coloured people as a coherent cultural identity, and not a social construction imposed by the apartheid regime, but to some individuals, the identity is imbued with shame and uneasiness about ethnicity (Hendricks 2001). The same author states that whatever one’s stance, it seems fair to say that the history of coloured identity has been heavily shaped, on the one hand, by racial oppression imposed by the nationalist regime, and, on the other hand, by the cultural creativity shown (in part, as a response) by coloured people themselves.

As an adult, I am constantly aware of how coloured people are perceived by society. On a recent trip to Johannesburg, I was involved in a meeting with a group of students. On the second day, one of the students asked, ‘Are you sure you are Coloured and from Cape Town?’ I responded, ‘Why?’ The person began to speak about a show performed by a popular South African comedian who is from a mixed-race family, and he even started to act out some of his sketches. I responded by saying that ‘we are not all like that…not all Coloured people speak like that’. The exaggerated coloured stereotype often portrayed in the media is of people with no front teeth and a ‘funny’ accent. As I reflect on my early childhood years, which were characterised by rampant racial discrimination, I remember the following incident.

#### ‘Whites Only’

I am 5 years old and we are standing on the train station on our way to hospital for my mom’s antenatal visit. A big maroon and yellow locomotive pulls into the platform. I indicate to my mom that I want to get into the carriage and she points to a board on the train that says ‘Whites Only/Net Blankes’. We walk to one end of the platform, to a carriage with a sign that says ‘Non-Whites/Nie Blankes’. Always obedient, I take hold of my mom’s hand as we board the train. The train ride was uncomfortable; the plastic seats were hard. However, I was now looking forward to the outing with my mom, and had forgotten the sign, and that we were not able to enter the ‘Whites Only/Net Blankes’ carriage. But later, the experience sparked many questions in my mind – ‘why were we not allowed on the train? What did “Whites Only” mean?’ To this day, the memory of my experiences of that day is still vivid in my mind.

In terms of identity, I see myself as a South African first, then a female and thereafter I am ‘statistically’ coloured. The colour of my skin in this democratic dispensation does not define me as a person, as I am free to assert my own humanity. However, as the reader of this article, you will recall that I said earlier on that I experience my coloured-ness as an unchangeable part of myself. All of the cultural meanings and stereotypes associated with the word ‘coloured’ imposed by the apartheid government are inescapable to me, as they are very much alive in my family and community. To me, being coloured is more than genetic makeup, racialised politics and culture; to me, it also signifies shame, poverty, exclusion and never having escaped the oppression of the apartheid regime. In 1983, Marietjie de Klerk, who would later be first lady to F.W. de Klerk, described the coloured community as ‘the people that were left after the nations were sorted out. They are the rest’ (Adendorf 2016).

### Theme 1: Children of Deaf adults as language brokers

More than describing who I am, the term ‘Mother father Deaf’ gives me access to the Deaf world. Recently, while passing a cell phone shop, I noticed a couple frantically signing away to the counter staff. I observed a need for assistance. Approaching the couple, I drew their attention by tapping one of them on the shoulder and greeted them with ‘Mother father Deaf, me hearing’. They immediately responded with a smile and asked me the name of my parents and what school they attended. These are important details during such an introduction. Thereafter, I asked if I could assist with interpreting and without hesitation they agreed. In this interaction, I was assuming the very familiar role of an interpreter and language broker, bridging a linguistic and cultural divide between the Deaf and hearing worlds, a role that I have played since my early childhood.

In a study of the life experiences of CODAs, Preston (1994) found both positive and negative accounts of what it is like to share in Deaf culture. In his analysis of gendered roles in CODAs, he identified that it is most often the eldest female child who takes on the role and responsibility of family interpreter. While my sister and I are both fluent signers, the role of interpreter was assumed by me. I presume that is what is common among CODAs; witnessing the breakdown of communication between Deaf and hearing worlds virtually compels one to take up the role of interpreter. But while CODAs are being praised for their roles within the family, a key question is how they, themselves, experience this, and how it impacts on the trajectory of their lives – issues which are rarely explored, hence my quest to pursue this auto-ethnographic study.

### Theme 2: Being bilingual and trilingual

My mother was my first teacher, and she exposed me to spoken language by using SASL and English together. She would mouth the English words, while also finger spelling, speaking and signing at the same time. I used SASL from early on in my life and I identify as a native signer. Having equivalent proficiency in each, I see SASL and English both as first languages, but still, SASL is our home language, while English is the first language of my hearing identity. My vocabulary was further complicated as I progressed from being bilingual to trilingual, when my mom began teaching me to speak Afrikaans, which was her family’s first language. It was also a language imposed on coloured people during the apartheid regime. Hence, I acquired three languages as a child. My mother has always been proud of the fact that I am fluent in three languages, and that I am able to interpret everything that is said in her presence.

Some studies show that children who grow up in bilingual settings are able to navigate two languages before the age of 2 (Deuchar & Quay 2000; Nicoladis & Genesee 1996). Napier, Rohan and Slayter (2005) suggest that the languages of bilinguals later alter with their evolving life circumstances. Hearing children born to Deaf parents are considered bilingual and bicultural, as they potentially share the language and culture of their Deaf parents. Singleton and Tittle (2000) point out that CODAs are also hearing individuals, and will inevitably acquire the dominant, spoken mode of communication and become members of the hearing community.

While I was growing up, my parents were referred to by others as ‘deaf and dumb’. I would often hear people refer to them as ‘dommies’ (dummies) – people who spoke broken English and had funny voices. Consequently, my sister and I were seen to have speech problems, and our hearing was tested regularly. I knew that behind the scenes, I was also referred to as ‘dumb’ – after all, that is how my own parents were viewed. I do not believe that I had significant problems with my speech development, but I do recall having a lisp and being teased for it. This, and the fear of being called dumb, led me to start practising my speech each day. I began to make sure that I pronounced each word in my head first before I uttered it. This had a profoundly negative effect, as I could hardly answer any questions in class for fear of mispronunciation or lisping, but my grades were always good. I would always first need to visualise the sign in my head before being able to utter the word from my mouth.

I often found it difficult to express myself and the same challenge has perpetuated into my adult years. I do not think my teachers ever noticed that I was fingerspelling words underneath my desk during tests. I would sign to myself, in order to make sense of what I was reading. Knowing sign language, it seems, was good for my grades! But it also caused me to turn my confidence inwards, rather than expressing it openly. I might know the word in my head, but my mouth simply would not utter it. On reflection, I attribute this scenario to what Burton (2015) describes as the presence of confidence but the absence of self-esteem, which in my case could have resulted from the stigma that I experienced because of the Deafness of my parents. As an adult, I find it difficult not to speak with my hands, as I feel that using spoken language is not enough – that my words are not expressive enough. Like a painter expressing myself on canvas, I need my audience to see what I am saying. Frank (2014) states that, because hearing children in Deaf families are typically visually oriented learners, processing information orally rather than visually may be a challenge for them.

Similar to the findings of a study undertaken by Singleton and Tittle (2000), from my early childhood years, my use of language has been changing along with a changing environment, or simply as the need arises. According to my mother, I was fluent in SASL by the age of 2, and by 4, I was a fully-fledged family interpreter, often filling the role of sole communicator with the outside world. I became the voice of the doctor, nurse, school teacher, social worker, grandparents and anyone else who could not communicate directly with my mother and father. Because of this, I was regularly exposed to situations that exceeded my level of maturity at any given time, forcing me to function as an adult while still a child. My usefulness as an interpreter depended on my having achieved fluency in SASL. According to research, when CODAs do not become fluent signers, their communication with their own parents is likely to be limited and ‘superficial’ (Hadjikakou et al. 2009).

### Theme 3: Being bicultural

In my ‘bicultural’ positioning as a CODA, I have had to recognise that I am not Deaf, but I am also not hearing. In addition, my racial identity is located, to some at least, on a borderline. All who grew up under apartheid were subject to a cultural and legislated set of racial descriptors which determined who, what and where one ought to be. As a consequence, I live with a complex pattern of difference, and it is this difference which I now try to examine in this auto-ethnographic study. Bull (1998) noted that the confirmation and acceptance of personal and cultural identity of CODAs may only occur in adulthood after encountering people with similar demographics. Until that point, CODAs may remain confronted with a split between two worlds, and in their own identity. Whether d/Deafness is defined on the basis of hearing ability or cultural affiliation, both definitions set up a dichotomy between h/Hearing and d/Deaf, such that in some instances, being one means not being the other (Pizer et al. 2015).

Preston (1994) noted that many CODAs only realise their difference at schoolgoing age – a realisation that probably signals the beginning of confusion or conflict regarding self-identity. Singleton and Tittle (2000) make the provocative statement that ‘Deaf parents are essentially raising “foreign” children’ (p. 27). The implication here is that, while parents are part of the Deaf community, their children are bicultural and bilingual, with access to cultural and community life outside of the ‘Deaf world’. Personally, I have always felt different, but the same. I grew up in two worlds – different to my parents, but bound by a culture which separated me from my peers, to whom I was, in turn, bound by hearing, and society’s definition of normalcy. Preston (1994) found that most CODAs understand deafness as a negative experience, and a disability, besides being a cultural minority. Having to traverse these different, and differently valued, worlds can create conflict and struggle in the children of Deaf parents.

### Theme 4: Role reversal and ‘parentification’

Always accompanying my mother wherever she went meant I seldom had the chance to be myself. My father converted the space in front of our door into a play area for me – that way, I was always nearby. I outline below the role reversal and parentification experiences I had with my mother; I reserve those that I shared with my father for another publication, in order not to take this article beyond its requirements in terms of length.

#### Fallopian tubes

I am 5 years old and my mother and I are at the hospital after the birth of my sister. The issue was that she had the option of undergoing sterilisation, and I had to interpret the conversation. Questions and answers flew, and while I also had to concentrate on allaying my mother’s fears, reassuring her that ‘it will all be ok’, some of the words were long, like ‘fallopian tubes’, and besides, I wanted to be playing with my dolls. But I had to focus – I was the only person my mother trusted. My role came with responsibility, but also with power, regardless of whether I wanted it or not. This reversal had a profound effect on my relationship with my parents. My parents played their role of providing well for my needs, but there were always situations in which I had to take charge, young as I was.

#### Ambulance

I was 12, and my 7-year-old sister was playing at a neighbour’s house. I heard someone shouting at the gate, saying that my sister was hurt. Her finger had been closed in a door, and was bleeding terribly. My first response was to tell my mother, then I ran to my sister’s side. Crying and in shock, I asked the neighbours to call an ambulance, as we did not have our own car to take her to the day hospital. When the ambulance arrived, we were told that only one person could accompany my little sister. Without hesitation, I climbed into the ambulance. There was no doubt that I had to be the mother; no doubt that it was my responsibility to make sure that my sister was taken care of. Even if my mother was able to accompany her, there would still be the challenge of communication barriers. She would struggle to communicate with the nursing staff – resulting in frustration, or even humiliation, in a health care environment which is ridden with numerous barriers, including non-sign language using staff; I had to protect my mother from this.

#### Terrible news

It is the weekend and our family home is unusually quiet. The mood is sombre; death is lurking on our doorstep. Unbeknown to my mother, my grandfather has been admitted to hospital after his fishing vessel capsized and he had nearly drowned. My mind is racing with thoughts about how my mother will react. My mother’s father is everything to her, but it is my responsibility to tell her what has happened, that her beloved father is gravely ill. I consider a dozen different ways to tell her; ‘Why must it be up to me? Why can’t her siblings break the news?’ I think about lying, and saying that he is just in hospital and will be ok. But then that is deceiving her, and she relies on me for the truth. My grandfather and I have always been close – my confidante and counsellor, and an important parental figure in my life. Inside, I am frantically praying that God will not take him away; there is no one who understands me like he does. But then I should not shed any tears, because any moment from now I could be called. As I enter the house, I look at my mother’s face, and I see she is looking confused and angry: ‘What is going on?’ she asks. I relay the message, my own emotions held at bay. My mother begins to cry hysterically, asking between sobs whether he will be ok, and saying that I have to tell her the truth.

Buchino (1993) describes role reversal as ‘when the child feels responsible for the parent and the parent expects them to be responsible’ (p. 44). However, it is important to note that parents are often concerned about the effect that the role of interpreter has on their hearing children (Torres 2003). The role that I assume as a CODA is one that silently speaks about protection and responsibility – I am constantly aware that nobody understands my parents. While CODAs are different to their parents in that they can hear and speak, respondents in a study undertaken in the USA among adult CODAs indicated that they are often disappointed by the common negative societal practices that marginalise them (Preston 1994). In other words, respondents felt the need to protect their parents against insults and negative views about Deaf people. Preston described them as ‘repositories of their hearing grandparents’ and parents’ untold stories’ (p. 67). There is also evidence of CODAs taking on parental responsibilities, such as looking after the finances, health and everyday challenges that a family faces (Orellana, Dorner & Pulido 2003), leading to opaque, co-dependent relationships and unclear family hierarchies.

#### Being a parental child in the face of apartheid

Returning to the issue of my coloured identity, my supervisor/co-author was curious about the interaction between caring for my parents’ vulnerabilities which are engendered by disablism, and those more based in apartheid’s structural racism. Subjectively, othering based on these different personal aspects is likely to merge and become indistinguishable. At the Coloured Affairs office, one is both coloured – with all that it meant in the South Africa of the 1980s – and disabled. In protecting my mother from the humiliations of disablist othering, I was also holding, for her, vulnerabilities to do with racism. My final anecdote brings these two devalued identities together. Part of what CODAs do, it seems, is to hold their parents through oppression. In my case, that oppression took at least two forms, leaving me, as a child, to somehow respond to or repair the contradictions of our distorted society.

### Theme 5: Issues of identity

Raised in both hearing and Deaf worlds, I was exposed to the politics and practices of both worlds. As I have said before, when introducing myself to Deaf people using SASL, I often say ‘Mother father Deaf, me hearing’ in order to gain acceptance. By contrast, when meeting hearing people for the first time, I do not introduce myself as a hearing child of Deaf parents. I just introduce myself as me – our shared normalcy, and our ‘fitting together’, is assumed. Enculturated into the Deaf world, I grew up part of the Deaf community, but in relation to education, communication and a host of forms of exclusion and discrimination, I do not have the same experience. My experience is not that of deafness, but of having the experience of my parents of deafness being somehow imposed on my life. Evidence shows that not all CODAs see themselves as being either hearing or Deaf; some regard themselves as being Deaf, even though they can hear (Preston 1994).

#### So… who am I?

So am I a hearing person raised in a Deaf community or a Deaf person trapped in a hearing body? I am somewhere in-between, holding onto conflicted emotions. I have known from a young age that my family is different, but I never saw myself as being different to my parents. I always believed that I was special as that was the response from the hearing world. People would make comments such as: ‘what a special girl she is’ or ‘you are so lucky’. Lucky to have Deaf parents or lucky that I was born with normal hearing? Are they suggesting that my parents are unlucky to not have normal hearing? I often wondered.

In school and meetings between my parents and teachers, I was the only child who had to interpret the meeting and not just deal with my academic progress. And then I had to deal with sympathy, beginning with the repeated ‘Oh shame!’ from parents of schoolmates. Despite the fact that I would have a packed lunch every day, my teachers would often share their lunch with me, saying, ‘it’s important for you to have a good meal every day’. I could not say No! Yes, we were poor and we lived in a wood and iron house without electricity, but the bottom line is that I was well taken care of. My understanding is that this ‘kindness’ was part of my family’s stigmatisation by the hearing community. We were seen as worthy of pity when, in fact, we were just like them, except we spoke with our hands. Negative attitudes towards my parents were also directed at me. Faced with this experience, I would wander between my two identities, feeling trapped on the periphery of both cultures. Yes, I may be hearing, but I am proudly Deaf, and then there is the little girl smothered inside.

## Conclusion

As noted above, the themes that emerged from this study are: (1) CODAs as language brokers, (2) being bilingual and trilingual, (3) being bicultural, (4) role reversal and parentification and (5) issues of identity. The themes show that the life world of a female, coloured CODA within a South African context is complex, multi-layered and multidimensional. The result is negative and positive experiences that are influenced at most by the intersection of various identity markers that include disability, gender, race, age, nationality, culture and language, within a synchronised hearing or Deaf world. As shown in the themes discussed above, and from as early as the age of 5 and as a CODA, Jane manages far more than just communication in her mediating role, as diverse situations often call upon her to contain a host of vulnerabilities that are lived not only by her, but also by her Deaf parents as well, in a scenario that is predominantly characterised by role reversal and parentification. She is charged with making sense of, or somehow digesting, the ills and inequalities of society, as these are channelled through the oppressive treatment of her Deaf parents, including in their effort to gain access to health care and social services.

As revealed by the discussion in Theme 4, the Department of Health in South Africa appears to offer blanket services that do not cater for the appropriate communication needs of Deaf adults. Consequently, CODAs who are as young as the age of 5 end up having to be interpreters and language brokers between their parents and health care staff, including in situations where sterilisation and fallopian tubes are discussed among adults. Such a scenario can be burdensome to young children, to the detriment of their psychosocial and general well-being. There is need to formulate policies that direct adult SASL interpreters in the form of family or community members to accompany Deaf people to service providers. But then again, some family members may be uninterested in learning to use sign language. The Department of Health should therefore consciously recruit and train Deaf people, thereby enabling them to bring positive change to service provision. In any case, a health care practice which is not multidimensional is likely to fall far short of reaching all vulnerable groups, including Deaf people.

Civil society, organisations for people with disabilities and adult CODAs need to work together to raise awareness regarding the complex location of CODAs within hearing or Deaf communities, thereby reducing obliviousness or unresponsiveness to the subject. Indifference may mean permissiveness or tolerance of ‘using’ minors as interpreters, language brokers and mediators in adult affairs, thereby sending a strong but inappropriate message to families, communities and various service sectors. Honikman et al. (2012) argue that raising children is a serious matter; hence, ‘burdening’ children with responsibilities that are not in sync with their age may result in compromised childhood development of CODAs. In any case, today’s children are the citizens of tomorrow’s world; their survival, protection and development is the prerequisite for future development and humanity. We call upon other scholars to undertake further research which locates the experience of CODAs within particular social and political contexts, thereby further contributing to this body of knowledge.

## Footnotes

1 While the term ‘coloured’ was part of the oppressive system of racial classification under apartheid, it has also been adopted by a large community of South Africans who self-identify as ‘coloured’ people. This community is concentrated in the Western Cape and Northern Cape provinces, and is largely Afrikaans speaking.
